# Causes of death in female patients with bladder cancer after local tumor excision and radical cystectomy: a contemporary, US population-based analysis

**DOI:** 10.1186/s40001-022-00873-y

**Published:** 2022-11-04

**Authors:** Qian Lyu, Yu Nie, Jiazheng Yuan, Dong Wang

**Affiliations:** Robot Minimally Invasive Center, Sichuan Academy of Medical Sciences and Sichuan Provincial People’s Hospital, Chengdu, 610072 China

**Keywords:** Bladder cancer, Women, Population-based, Local tumor excision, Radical cystectomy, Prognosis

## Abstract

Surgery is one of the most important treatments for bladder cancer, including local tumor excision and radical cystectomy. At present, studies on the causes of death for contemporary survivors, especially women, who have received different surgical treatments are limited. Therefore, the study used a population-based cohort study in the United States from 2000 to 2017 to analyze causes of death for women who underwent local tumor excision or radical cystectomy stratified by demographics and tumor stage. standardized mortality ratios (SMRs) were calculated based on general population data. In total, 24,040 female patients who underwent surgical treatments were assessed. Of those 20,780 patients undergoing local tumor excision, 36.6% died of bladder cancer, while 63.4% died of other causes. The risk of death from all causes increased in comparation with the general population (SMR 1.85; 95% CI 1.82–1.87), and the most common non-tumor cause of death was from heart diseases (16.2%; SMR 1.13; 95% CI 1.09–1.16). Among women who receive radical cystectomy, 82.3% of deaths occurred within 5 years after surgery. 66.9% deaths resulted from bladder cancer, and the risk of death from all causes significantly higher than that in the general people (SMR 4.67; 95% CI 4.51–4.84). Moreover, the risk of death from non- bladder cancer causes also increased, in particular, such as septicemia (SMR 3.09; 95% CI 2.13–4.34). Causes of death during bladder cancer survivorship after surgery vary by patient and tumor characteristics, and these data provide information regarding primary care for women during postoperative cancer survivorship.

## Introduction

Bladder cancer is one of the most common malignant tumors and the incidence increases steadily worldwide. More than 500,000 new cases have been confirmed, which accounts for about 3% of all new cancer diagnoses each year, and 200,000 deaths worldwide [1]. Previous study has suggested a positive association between the bladder cancer incidence and human development index and gross domestic product [2]. Metabolic syndrome (MS), a non-negligible public health problem, is characterized by lipid disorders, abnormal glucose tolerance, high blood pressure, and a high mortality rate. The disorder has been reported to be associated with the development and the risk of death of bladder cancer. A retrospective study [3] that involved 169 patients suggested that patients with MS had a higher histological grade of bladder cancer, as well as the low high-density lipoprotein levels. The high body mass index (BMI) has also been considered to be associated with the risk of bladder cancer [4]. In the American population, more than 80,000 new cases are diagnosed each year, representing 4.6% of all cancer diagnoses, which is greater than global average. Statistics showed that about 17,900 US patients died of bladder cancer in 2019 [5]. Across the world, the number of men diagnosed with bladder cancer is about four times that of women, and the mortality rate is similar [6]. However, studies have shown that for patients with the same stage of bladder cancer, the prognosis of women is worse than that of men [7]. The occurrence of bladder cancer is a complex, multifactorial and multi-step pathological process, which is affected by both internal genetic factors and external environmental factors. Hence, bladder cancer contains of various pathological types and complex treatment modalities. However, for the majority of patients, surgery is still the main means of treatment, including transurethral resection of bladder tumor, partial cystectomy and radical cystectomy etc. [8]. Different surgical methods should be rigorously determined according to the pathological results and the grading and staging of the disease, because of significant differences of the quality of life and prognosis of patients. Hence, understanding the actual causes of death in contemporary bladder cancer cases undergoing different surgical methods can help with a more rigorous surgical plan and proper health care during survivorship.

Several previous studies have illustrated the causes of bladder cancer-specific mortality [9–11], however, the information about causes of death in patients with bladder cancer after local tumor excision and radical cystectomy are limited. Simultaneously, most studies concentrate on patients of all genders, and few studies pay attention to specific gender, especially female patients. Factors such as hormone level, lifestyle, occupational exposure vary widely in male and female population, which may result in differences on causes of death. Hence, understanding the information could guiding the long-term follow-up and therapeutic strategies, and we evaluated contemporary, female population-based data for causes of death during bladder cancer after local tumor excision or radical cystectomy survivorship in the United States using SEER database.

## Materials and methods

### Data source

The data were acquired from Surveillance, Epidemiology, and End Results (SEER) program which is conducted by National Cancer Institute covering approximately 48% of the US population, and the database SEER 18 registries were accessed from 2000 to 2017 using the SEER ∗ Stat software 8.3.8. The data used are publicly available and our study did not require a declaration or approval of local ethics.

### Study population

We included all female patients with a diagnosis of bladder cancer between January 1, 2000, and December 31, 2017 in US, and only first malignant neoplasm was selected. Simultaneously, we excluded data without surgical treatment. We also exclude patients diagnosed only through death certificate and autopsy, patients with unknown follow-up time, survival status, and reasons of death, and patients without general information including age and race.

### SMR

The number of deaths in different variables was measured for patients with bladder cancer from the SEER database. Patients were mainly assessed by different surgical methods including local tumor excision and radical cystectomy, and then, stratified by age, year of diagnosis, race, tumor differentiation, and pathological type. All causes of deaths were considered in our study, we divided the causes into malignant cancer group, non-tumor group. Under diseases of the malignant cancer, we included the most common malignant diseases of digestive system, respiratory system, female genital system, urinary system, and lymphatic system. In the non-tumor group, simultaneously, we included virus systematic disorders, such as septicemia, other Infectious and Parasitic Diseases including HIV, diabetes mellitus, Alzheimer’s, diseases of heart, hypertension without heart disease, cerebrovascular diseases, other diseases of arteries, arterioles, capillaries, pneumonia and Influenza, and chronic obstructive pulmonary disease and allied cond. We counted the numbers of deaths in different subgroups at each follow-up stage, and calculated SMR, the ratio of observed-to-expected, with 95% confidence intervals for each cause of death after bladder cancer diagnosis undergoing different surgical treatments. From 2000 to 2016, female patients diagnosed with bladder cancer and underwent surgical treatment constituted the observed population, while the expected population consisted of the general population who were diagnosed between 1975 and 2016, and the data were collected from the SEER database.

### Statistical analysis

We calculated SMRs with 95% confidence intervals using the SEER ∗ Stat software 8.3.8 (https://seer.cancer.gov/seerstat/software/). The higher number of deaths with bladder cancer than the expected number in the general population was regarded as a significantly increased risk. *p*-value < 0.05 was considered to be statistically significant.

## Results

### Baseline characteristics

24,040 female patients with bladder cancer undergoing surgical treatments were collected in our study, in which 20,780 patients received local tumor excision, and 3260 underwent radical cystectomy. Table 1 details the number and SMR with 95% CI of patients by age, year of diagnosis, race, tumor differentiation, pathological type and time period for all deaths by each grouping. The total excess risk of local tumor excision group was 353.65 per 10,000, while 1137.11 in the radical cystectomy group.

**Table 1:** Baseline characteristics of patients with bladder cancer after local tumor excision and radical cystectomy

Variables	Total				2–11 months				12–59 months				60–119 months				120 + months			
	Observed	Persons	Excess Risk	SMR (95%CI)	Observed	Persons	Excess Risk	SMR (95%CI)	Observed	Persons	Excess Risk	SMR (95%CI)	Observed	Persons	Excess Risk	SMR (95%CI)	Observed	Persons	Excess Risk	SMR (95%CI)
Local tumor excision																				
All	20,780	45,655	353.65	1.85^#^ (1.82–1.87)	5453	45,655	1211.82	4.18^#^ (4.07–4.29)	8096	38,024	302.9	1.77^#^ (1.73–1.81)	4694	22,279	152.66	1.35^#^ (1.31–1.39)	2537	10,519	162.7	1.34^#^ (1.28–1.39)
Age, year																				
15–54 years	782	4997	133.31	3.85^#^ (3.58–4.13)	226	4997	543.03	19.94^#^ (17.42–22.71)	291	4557	150.26	5.33^#^ (4.74–5.98)	153	3409	61.87	2.29^#^ (1.95–2.69)	112	2177	42.33	1.59^#^ (1.31–1.91)
55–64 years	1987	8405	198.84	2.66^#^ (2.54–2.77)	450	8405	609.53	9.25^#^ (8.42–10.15)	703	7531	193.42	3.17^#^ (2.94–3.41)	489	5131	124.06	1.99^#^ (1.82–2.17)	345	2800	100.67	1.49^#^ (1.34–1.65)
65–74 years	4684	12,338	289.68	1.95^#^ (1.89–2)	971	12,338	849.05	5.88^#^ (5.52–6.26)	1663	10,713	279.28	2.30^#^ (2.19–2.42)	1142	6583	157.82	1.49^#^ (1.41–1.58)	908	3185	133.06	1.20^#^ (1.13–1.29)
75–84 years	8018	13,035	427.11	1.53^#^ (1.49–1.56)	1927	13,035	1524.27	4.15^#^ (3.96–4.33)	3088	10,553	317.92	1.47^#^ (1.42–1.52)	2042	5620	74.6	1.07^#^ (1.03–1.12)	961	2045	309.55	1.24^#^ (1.16–1.32)
85 + years	5306	6860	1332.18	2.00^#^ (1.95–2.06)	1878	6860	2752.94	3.05^#^ (2.91–3.19)	2349	4652	797.49	1.60^#^ (1.53–1.66)	868	1521	977.88	1.75^#^ (1.63–1.87)	211	303	2704.32	3.13^#^ (2.72–3.58)
Year of diagnosis																				
2000–2007	11,960	19,150	288.4	1.67^#^ (1.64–1.7)	2326	19,150	1181.94	3.98^#^ (3.82–4.14)	3982	16,785	291.86	1.73^#^ (1.68–1.78)	3163	12,715	135.85	1.31^#^ (1.26–1.35)	2489	9398	158.96	1.33^#^ (1.28–1.38)
2008–2012	5426	12,194	367.15	1.92^#^ (1.87–1.97)	1453	12,194	1176.14	4.16^#^ (3.95–4.38)	2444	10,688	277.41	1.72^#^ (1.65–1.79)	1481	8090	186.85	1.43^#^ (1.36–1.51)	48	1121	428.7	1.94^#^ (1.43–2.57)
2013–2017	3233	12,273	628.45	2.66^#^ (2.56–2.75)	1513	12,273	1253.43	4.41^#^ (4.19–4.63)	1670	10,551	374.34	1.98^#^ (1.88–2.07)	50	1474	298.36	1.73^#^ (1.28–2.28)	0	0	0	0 (0–0)
Race																				
White	18,245	40,410	325.72	1.77^#^ (1.74–1.8)	4637	40,410	1138.43	3.95^#^ (3.83–4.06)	7046	33,881	275.42	1.69^#^ (1.65–1.73)	4230	20,212	141.37	1.32^#^ (1.28–1.36)	2332	9648	156.81	1.32^#^ (1.27–1.37)
Black	1810	3254	792.24	2.83^#^ (2.7–2.96)	624	3254	2298.18	6.68^#^ (6.17–7.23)	746	2481	683.25	2.65^#^ (2.46–2.85)	318	1193	343.03	1.74^#^ (1.56–1.95)	122	476	233.05	1.49^#^ (1.24–1.78)
American Indian/Alaska Native	48	107	710.02	4.71^#^ (3.47–6.25)	14	107	1601.65	9.70^#^ (5.3–16.28)	25	86	954.66	6.74^#^ (4.36–9.94)	2	41	− 82.54	0.61 (0.07–2.22)	7	24	661.59	3.94^#^ (1.58–8.12)
Asian or Pacific Islander	677	1884	367.07	2.26^#^ (2.1–2.44)	178	1884	995.92	4.89^#^ (4.2–5.67)	279	1576	335.5	2.24^#^ (1.98–2.51)	144	833	187.63	1.61^#^ (1.36–1.9)	76	371	200.85	1.56^#^ (1.23–1.95)
Differentiation																				
Well-differentiated	2425	6461	75.08	1.20^#^ (1.15–1.25)	214	6461	91.32	1.29^#^ (1.12–1.47)	864	6205	54.71	1.16^#^ (1.08–1.24)	801	4596	74.27	1.19^#^ (1.11–1.27)	546	2446	113.63	1.25^#^ (1.15–1.36)
Moderately differentiated	5288	13,730	117.98	1.30^#^ (1.26–1.33)	639	13,730	248.61	1.76^#^ (1.62–1.9)	1850	13,009	81.56	1.23^#^ (1.17–1.28)	1695	8245	105.66	1.25^#^ (1.19–1.31)	1104	4617	144.5	1.31^#^ (1.23–1.38)
Poorly differentiated	4902	7218	789.82	2.60^#^ (2.53–2.68)	1693	7218	2836.00	6.96^#^ (6.63–7.3)	1882	5479	691.45	2.46^#^ (2.35–2.57)	878	3119	285.37	1.57^#^ (1.47–1.67)	449	1537	229.89	1.42^#^ (1.29–1.56)
Undifferentiated	5682	10,018	1,003.81	3.04^#^ (2.96–3.12)	2216	10,018	2634.29	6.64^#^ (6.36–6.92)	2465	7717	824.37	2.74^#^ (2.63–2.85)	745	2857	307.42	1.59^#^ (1.48–1.71)	256	1007	313.72	1.54^#^ (1.35–1.74)
Unknown	2322	6190	263.09	1.69^#^ (1.62–1.75)	530	6190	740.43	3.11^#^ (2.85–3.39)	1035	5614	216.31	1.59^#^ (1.5–1.69)	575	3462	149.68	1.36^#^ (1.25–1.48)	182	912	154.56	1.35^#^ (1.16–1.56)
Pathological type																				
8130/3: papillary transitional cell carcinoma	13,058	34,058	171.44	1.42^#^ (1.39–1.44)	1888	34,058	346.59	1.96^#^ (1.87–2.05)	5254	30,448	161.19	1.42^#^ (1.38–1.46)	3774	18,797	127.75	1.30^#^ (1.26–1.34)	2142	9041	149.73	1.31^#^ (1.25–1.36)
8120/3: transitional cell carcinoma, NOS	6089	9282	1190.93	3.51^#^ (3.42–3.6)	2660	9282	3861.82	9.18^#^ (8.84–9.54)	2328	6243	960.35	3.12^#^ (2.99–3.25)	775	2870	301.13	1.60^#^ (1.49–1.72)	326	1183	257.41	1.51^#^ (1.35–1.68)
8070/3: squamous cell carcinoma, NOS	421	475	5377.79	13.35^#^ (12.11–14.69)	296	475	13,208.44	27.42^#^ (24.38–30.72)	100	168	2778.97	7.56^#^ (6.16–9.2)	21	47	1160.00	4.02^#^ (2.49–6.15)	4	14	284.77	1.75 (0.48–4.47)
8010/3: carcinoma, NOS	166	262	959.88	3.13^#^ (2.68–3.65)	78	262	4175.02	10.86^#^ (8.58–13.55)	55	172	700.93	2.73^#^ (2.05–3.55)	23	96	128.36	1.26 (0.8–1.88)	10	42	184.47	1.37 (0.66–2.52)
8140/3: adenocarcinoma, NOS	162	250	1436.30	6.02^#^ (5.13–7.02)	65	250	3645.14	12.89^#^ (9.95–16.43)	74	172	1493.70	6.89^#^ (5.41–8.65)	15	73	321.54	2.07^#^ (1.16–3.41)	8	27	369.81	2.07 (0.89–4.08)
8041/3: small cell carcinoma, NOS	124	169	2688.94	6.60^#^ (5.49–7.87)	74	169	7167.67	16.55^#^ (12.99–20.77)	40	84	1758.03	4.96^#^ (3.55–6.76)	7	29	236.32	1.44 (0.58–2.97)	3	8	738.72	2.14 (0.44–6.26)
8071/3: squamous cell carcinoma, keratinizing, NOS	117	129	6735.71	15.67^#^ (12.96–18.78)	94	129	17,566.75	35.52^#^ (28.71–43.47)	16	33	2062.71	5.26^#^ (3–8.53)	6	12	1339.84	3.91^#^ (1.44–8.52)	1	4	523	4.1 (0.1–22.87)
8050/3: papillary carcinoma, NOS	106	214	196.02	1.48^#^ (1.21–1.79)	20	214	818.66	3.23^#^ (1.97–4.99)	36	194	192.87	1.53^#^ (1.07–2.11)	26	133	59.45	1.14 (0.75–1.68)	24	94	125.65	1.26 (0.81–1.88)
Radical cystectomy																				
All	3260	5385	1137.11	4.68^#^ (4.52–4.84)	1155	5385	2778.17	13.07^#^ (12.32–13.84)	1526	3957	1259.28	5.68^#^ (5.4–5.97)	376	1730	264.95	1.73^#^ (1.56–1.91)	203	773	294.24	1.65^#^ (1.43–1.9)
Age, year																				
15–54 years	375	766	854.88	19.11^#^ (17.22–21.15)	125	766	2152.25	71.35^#^ (59.39–85.01)	201	605	1190.37	33.69^#^ (29.19–38.68)	34	305	231.88	5.55^#^ (3.85–7.76)	15	179	123.95	2.59^#^ (1.45–4.28)
55–64 years	669	1281	1009.47	9.93^#^ (9.19–10.71)	216	1,281	2232.86	30.79^#^ (26.82–35.18)	352	994	1,282.28	15.40^#^ (13.84–17.1)	66	457	271.16	3.14^#^ (2.43–3.99)	35	226	232.02	2.12^#^ (1.48–2.95)
65–74 years	1089	1801	1215.39	5.24^#^ (4.93–5.56)	372	1,801	2695.54	16.37^#^ (14.75–18.12)	523	1,328	1391.19	7.56^#^ (6.93–8.24)	116	549	306.46	1.95^#^ (1.61–2.34)	78	237	253.67	1.38^#^ (1.09–1.72)
75–84 years	965	1337	1337.07	2.84^#^ (2.67–3.03)	365	1337	3544.89	8.90^#^ (8.01–9.87)	389	913	1100.31	2.80^#^ (2.52–3.09)	144	384	221.93	1.22^#^ (1.03–1.44)	67	121	807.78	1.62^#^ (1.25–2.05)
85 + years	160	196	2044.26	2.54^#^ (2.16–2.97)	77	196	5197.02	4.84^#^ (3.82–6.05)	59	113	1152.30	1.86^#^ (1.42–2.4)	16	34	315.24	1.24 (0.71–2.02)	8	9	2,665.53	3.16^#^ (1.36–6.22)
Year of diagnosis																				
2000–2007	1758	2275	983.27	3.85^#^ (3.67–4.03)	543	2275	3056.31	13.24^#^ (12.15–14.4)	748	1727	1261.04	5.34^#^ (4.97–5.74)	266	965	272.26	1.72^#^ (1.52–1.94)	201	685	297.14	1.66^#^ (1.44–1.9)
2008–2012	887	1434	1200.85	5.41^#^ (5.06–5.77)	328	1434	2905.97	14.02^#^ (12.54–15.62)	453	1098	1211.21	5.75^#^ (5.23–6.31)	104	627	237.05	1.72^#^ (1.41–2.08)	2	88	121.81	1.38 (0.17–4.98)
2013–2017	596	1424	1631.79	8.00^#^ (7.37–8.67)	265	1424	2293.54	11.84^#^ (10.46–13.36)	325	1132	1326.86	6.50^#^ (5.81–7.24)	6	138	591.62	2.82^#^ (1.04–6.14)	0	0	0	0 (0–0)
Race																				
White	2750	4541	1108.75	4.43^#^ (4.26–4.6)	956	4541	2707.18	12.37^#^ (11.6–13.18)	1282	3361	1222.55	5.37^#^ (5.08–5.68)	330	1481	261.54	1.68^#^ (1.51–1.88)	182	658	316.37	1.66^#^ (1.43–1.92)
Black	365	557	1464.79	6.39^#^ (5.75–7.08)	139	557	3365.11	16.23^#^ (13.64–19.16)	178	389	1724.63	7.96^#^ (6.83–9.22)	33	155	331.37	2.14^#^ (1.48–3.01)	15	71	150.01	1.39 (0.78–2.29)
American Indian/Alaska Native	11	21	1554.96	19.42^#^ (9.69–34.74)	7	21	4314.66	41.26^#^ (16.59–85.01)	3	14	718.68	10.48^#^ (2.16–30.62)	0	5	-81.03	0 (0–34.78)	1	1	23,765.73	224.52^#^ (5.68–1250.94)
Asian or Pacific Islander	134	266	989.52	7.26^#^ (6.08–8.59)	53	266	2665.39	22.47^#^ (16.83–29.4)	63	193	1089.87	8.24^#^ (6.33–10.54)	13	89	223.1	2.20^#^ (1.17–3.76)	5	43	160.87	1.95 (0.63–4.56)
Differentiation																				
Well-differentiated	39	58	930.35	4.13^#^ (2.94–5.65)	12	58	2578.68	13.20^#^ (6.82–23.06)	15	46	822.44	4.16^#^ (2.33–6.86)	11	27	786.27	3.21^#^ (1.6–5.75)	1	9	-125.31	0.67 (0.02–3.72)
Moderately differentiated	245	382	1095.85	5.31^#^ (4.67–6.02)	87	382	2963.45	16.48^#^ (13.2–20.32)	111	294	1183.86	6.34^#^ (5.21–7.63)	33	145	375.09	2.34^#^ (1.61–3.28)	14	74	194.28	1.52 (0.83–2.55)
Poorly differentiated	1245	1731	1235.17	4.81^#^ (4.55–5.09)	445	1731	3367.21	15.09^#^ (13.72–16.55)	565	1275	1446.01	6.14^#^ (5.64–6.67)	143	607	275.36	1.75^#^ (1.48–2.06)	92	318	291.88	1.66^#^ (1.34–2.04)
Undifferentiated	1560	2692	1088.28	4.41^#^ (4.2–4.64)	540	2692	2467.14	11.41^#^ (10.47–12.41)	764	2126	1192.41	5.34^#^ (4.97–5.74)	172	851	220.4	1.57^#^ (1.34–1.82)	84	329	289.44	1.58^#^ (1.26–1.95)
Unknown	152	270	998.55	5.41^#^ (4.59–6.34)	52	270	2425.40	13.81^#^ (10.32–18.11)	71	216	1012.22	5.61^#^ (4.38–7.08)	17	100	255.57	2.03^#^ (1.18–3.24)	12	43	677.04	3.67^#^ (1.89–6.4)
Pathological type																				
8120/3: transitional cell carcinoma, NOS	1813	2906	1222.50	4.74^#^ (4.52–4.96)	637	2906	2846.27	12.88^#^ (11.9–13.92)	873	2127	1393.34	5.95^#^ (5.56–6.35)	195	885	249.03	1.64^#^ (1.42–1.89)	108	398	296.68	1.60^#^ (1.32–1.94)
8130/3: papillary transitional cell carcinoma	767	1449	780.42	3.45^#^ (3.21–3.7)	212	1449	1732.85	8.33^#^ (7.24–9.53)	369	1154	905.2	4.27^#^ (3.84–4.73)	122	561	268.95	1.73^#^ (1.44–2.06)	64	246	277.35	1.60^#^ (1.23–2.05)
8070/3: squamous cell carcinoma, NOS	219	324	1475.83	6.59^#^ (5.75–7.53)	116	324	5319.09	27.88^#^ (23.04–33.44)	74	200	1186.43	5.96^#^ (4.68–7.48)	17	98	182.44	1.59 (0.93–2.55)	12	45	330.84	2.01^#^ (1.04–3.51)
8071/3: squamous cell carcinoma, keratinizing, NOS	73	118	1335.20	5.66^#^ (4.44–7.12)	42	118	5275.67	24.35^#^ (17.55–32.91)	22	72	869.1	4.19^#^ (2.63–6.35)	5	37	119.05	1.41 (0.46–3.29)	4	14	274.06	1.68 (0.46–4.31)
8122/3: transitional cell carcinoma, spindle cell	68	104	2379.77	10.05^#^ (7.8–12.74)	43	104	6400.21	32.04^#^ (23.19–43.16)	19	53	1383.53	6.48^#^ (3.9–10.12)	5	19	464.74	2.5 (0.81–5.84)	1	6	440.6	2.02 (0.05–11.27)
8140/3: Adenocarcinoma, NOS	61	89	1301.96	7.40^#^ (5.66–9.5)	17	89	2396.29	15.22^#^ (8.87–24.37)	30	65	1526.49	10.34^#^ (6.98–14.76)	8	29	543.81	3.72^#^ (1.61–7.34)	6	16	728.82	2.88^#^ (1.06–6.27)

*SMR* standardized mortality ratio, *CI* confidence interval, *NOS* not otherwise specified

Excess risk is per 10,000

^#^Statistical significance with *P* < 0.05

### Causes of death for female patients undergoing local tumor excision

**Fig. 1:**
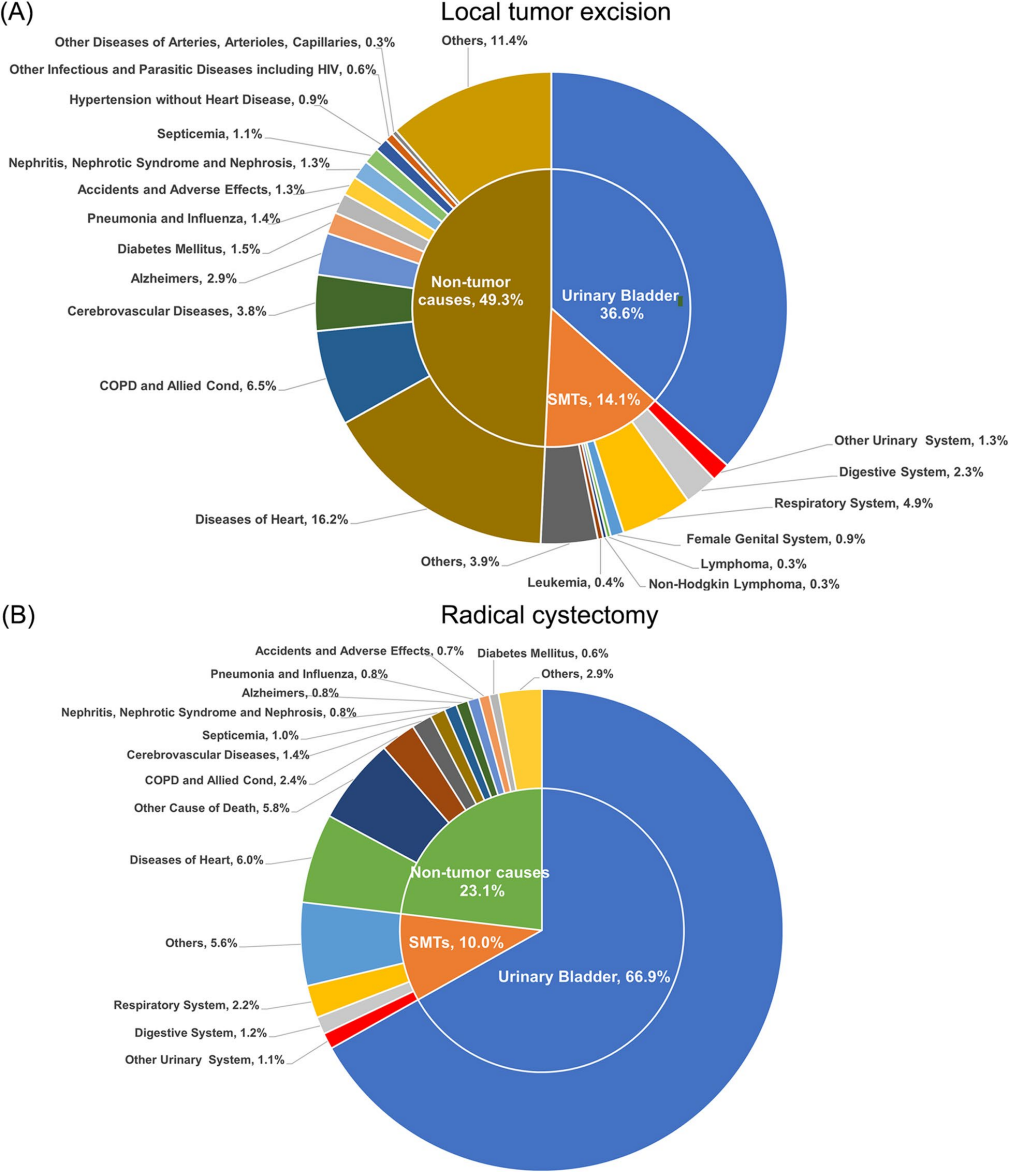
Proportion of different causes of death after operation, (**A**) Local tumor excision (**B**) Radical cystectomy

The majority of deaths for women undergoing local tumor excision occurred in12 to 59 months after surgery (*n* = 8096 [39%]), Table 1). In this group, as shown in Table 2 and Fig. 1, deaths from bladder cancer accounted for 36.6% of the all deaths (*n* = 20,780), which maintained a relative stable over the different follow-up periods. Deaths from other malignant cancers and non-tumor factors were 2933 and 10,239, respectively. In this study, the most common non tumor cause of death was diseases of heart, and the number of which was 3359, accounting 16.2% of all deaths, while in the malignant cancer group, cancers of lung and bronchus composed the majority, and the number was 1102, which accounted 5.3% of all deaths. Although the risk of death significantly decreased after 2–11 month follow-up (SMR 4.18; 95% CI 4.07–4.29), the risk was higher than that of general population over the follow-up months (SMR 1.77; 95% CI 1.73–1.81 over 12–59 months, SMR 1.35; 95% CI 1.31–1.39 over 12–59 months and SMR 1.34; 95% CI 1.28–1.39 over 60 months).

**Table 2:** Main causes of death for patients with bladder cancer after local tumor excision

	Total			2–11 months			12–59 months			60–119 months			120 + months		
	Observed	Expected	SMR (95%CI)	Observed	Expected	SMR (95%CI)	Observed	Expected	SMR (95%CI)	Observed	Expected	SMR (95%CI)	Observed	Expected	SMR (95%CI)
All causes of death	20,780	11,255.35	1.85^#^ (1.82–1.87)	5453	1305.87	4.18^#^ (4.07–4.29)	8096	4569.39	1.77^#^ (1.73–1.81)	4694	3479.86	1.35^#^ (1.31–1.39)	2537	1900.22	1.34^#^ (1.28–1.39)
All malignant cancers	10,541	1953.59	5.40^#^ (5.29–5.5)	4031	238.49	16.90^#^ (16.38–17.43)	4311	822.82	5.24^#^ (5.08–5.4)	1540	589.96	2.61^#^ (2.48–2.74)	659	302.33	2.18^#^ (2.02–2.35)
Digestive system	473	478.56	0.99 (0.9–1.08)	59	58.4	1.01 (0.77–1.3)	164	200.81	0.82^#^ (0.7–0.95)	160	144.63	1.11 (0.94–1.29)	90	74.71	1.2 (0.97–1.48)
Stomach	42	32.58	1.29 (0.93–1.74)	8	4.21	1.9 (0.82–3.74)	16	14.03	1.14 (0.65–1.85)	11	9.63	1.14 (0.57–2.04)	7	4.72	1.48 (0.6–3.06)
Colon and rectum	141	195.19	0.72^#^ (0.61–0.85)	12	24.6	0.49^#^ (0.25–0.85)	49	83.18	0.59^#^ (0.44–0.78)	47	58.29	0.81 (0.59–1.07)	33	29.12	1.13 (0.78–1.59)
Liver and intrahepatic bile duct	55	50.64	1.09 (0.82–1.41)	9	5.85	1.54 (0.7–2.92)	19	20.67	0.92 (0.55–1.44)	15	15.6	0.96 (0.54–1.59)	12	8.52	1.41 (0.73–2.46)
Liver	36	32.91	1.09 (0.77–1.51)	9	3.86	2.33^#^ (1.07–4.43)	11	13.53	0.81 (0.41–1.46)	9	10.09	0.89 (0.41–1.69)	7	5.44	1.29 (0.52–2.65)
Pancreas	167	143.47	1.16 (0.99–1.35)	22	16.92	1.3 (0.81–1.97)	60	59.31	1.01 (0.77–1.3)	63	43.95	1.43^#^ (1.1–1.83)	22	23.29	0.94 (0.59–1.43)
Respiratory system	1010	500.79	2.02^#^ (1.89–2.15)	62	61.7	1 (0.77–1.29)	443	213.38	2.08^#^ (1.89–2.28)	332	150.75	2.20^#^ (1.97–2.45)	173	74.96	2.31^#^ (1.98–2.68)
Lung and bronchus	1002	493.47	2.03^#^ (1.91–2.16)	60	60.79	0.99 (0.75–1.27)	438	210.25	2.08^#^ (1.89–2.29)	332	148.57	2.23^#^ (2–2.49)	172	73.86	2.33^#^ (1.99–2.7)
Breast	130	251.34	0.52^#^ (0.43–0.61)	10	31.04	0.32^#^ (0.15–0.59)	37	106.3	0.35^#^ (0.25–0.48)	52	75.44	0.69^#^ (0.51–0.9)	31	38.56	0.8 (0.55–1.14)
Female genital system	188	181.49	1.04 (0.89–1.2)	31	22.31	1.39 (0.94–1.97)	66	76.83	0.86 (0.66–1.09)	59	54.57	1.08 (0.82–1.39)	32	27.77	1.15 (0.79–1.63)
Ovary	103	94.25	1.09 (0.89–1.33)	15	11.82	1.27 (0.71–2.09)	35	40.51	0.86 (0.6–1.2)	34	28.16	1.21 (0.84–1.69)	19	13.76	1.38 (0.83–2.16)
Urinary system	7876	79.66	98.87^#^ (96.7–101.08)	3663	9.36	391.55^#^ (378.97–404.44)	3253	32.77	99.26^#^ (95.88–102.73)	730	24.47	29.84^#^ (27.71–32.08)	230	13.07	17.60^#^ (15.4–20.03)
Urinary bladder	7608	40.22	189.17^#^ (184.94–193.47)	3578	4.64	770.87^#^ (745.81–796.55)	3138	16.35	191.98^#^ (185.32–198.81)	683	12.44	54.89^#^ (50.85–59.16)	209	6.79	30.80^#^ (26.76–35.27)
Kidney and renal pelvis	153	36.45	4.20^#^ (3.56–4.92)	42	4.37	9.61^#^ (6.92–12.98)	60	15.21	3.94^#^ (3.01–5.08)	37	11.09	3.34^#^ (2.35–4.6)	14	5.77	2.42^#^ (1.33–4.07)
Ureter	37	1.6	23.09^#^ (16.26–31.82)	12	0.18	66.30^#^ (34.26–115.81)	21	0.65	32.34^#^ (20.02–49.44)	3	0.5	6.02^#^ (1.24–17.58)	1	0.27	3.66 (0.09–20.37)
Other urinary organs	78	1.39	56.04^#^ (44.3–69.94)	31	0.16	193.65^#^ (131.58–274.87)	34	0.57	60.00^#^ (41.55–83.84)	7	0.43	16.24^#^ (6.53–33.47)	6	0.23	25.62^#^ (9.4–55.76)
Lymphoma	56	81.42	0.69^#^ (0.52–0.89)	5	9.96	0.5 (0.16–1.17)	20	34.26	0.58^#^ (0.36–0.9)	21	24.58	0.85 (0.53–1.31)	10	12.62	0.79 (0.38–1.46)
Non-Hodgkin lymphoma	55	78.42	0.70^#^ (0.53–0.91)	5	9.58	0.52 (0.17–1.22)	20	32.98	0.61^#^ (0.37–0.94)	20	23.69	0.84 (0.52–1.3)	10	12.17	0.82 (0.39–1.51)
Leukemia	75	76.48	0.98 (0.77–1.23)	3	9.06	0.33^#^ (0.07–0.97)	30	31.64	0.95 (0.64–1.35)	28	23.45	1.19 (0.79–1.73)	14	12.34	1.13 (0.62–1.9)
Miscellaneous malignant cancer	592	153.5	3.86^#^ (3.55–4.18)	180	18.71	9.62^#^ (8.27–11.13)	258	64.33	4.01^#^ (3.54–4.53)	106	46.35	2.29^#^ (1.87–2.77)	48	24.11	1.99^#^ (1.47–2.64)
Non-tumor deaths															
Septicemia	231	162.89	1.42^#^ (1.24–1.61)	48	19.34	2.48^#^ (1.83–3.29)	73	67.06	1.09 (0.85–1.37)	74	49.92	1.48^#^ (1.16–1.86)	36	26.57	1.36 (0.95–1.88)
Other infectious and parasitic diseases including HIV	119	74.41	1.60^#^ (1.32–1.91)	30	8.37	3.58^#^ (2.42–5.11)	50	30.43	1.64^#^ (1.22–2.17)	29	23.59	1.23 (0.82–1.77)	10	12.02	0.83 (0.4–1.53)
Diabetes mellitus	307	280.55	1.09 (0.98–1.22)	59	35.03	1.68^#^ (1.28–2.17)	104	118.62	0.88 (0.72–1.06)	85	84.16	1.01 (0.81–1.25)	59	42.75	1.38^#^ (1.05–1.78)
Alzheimer’s	598	754.66	0.79^#^ (0.73–0.86)	40	76.55	0.52^#^ (0.37–0.71)	167	284.08	0.59^#^ (0.5–0.68)	204	242.72	0.84^#^ (0.73–0.96)	187	151.31	1.24^#^ (1.07–1.43)
Diseases of heart	3359	2984.53	1.13^#^ (1.09–1.16)	529	360.23	1.47^#^ (1.35–1.6)	1289	1231.38	1.05 (0.99–1.11)	995	909.16	1.09^#^ (1.03–1.16)	546	483.77	1.13^#^ (1.04–1.23)
Hypertension without heart disease	189	166.51	1.14 (0.98–1.31)	29	18.19	1.59^#^ (1.07–2.29)	63	65.34	0.96 (0.74–1.23)	59	52.87	1.12 (0.85–1.44)	38	30.12	1.26 (0.89–1.73)
Cerebrovascular diseases	796	824.92	0.96 (0.9–1.03)	114	99.72	1.14 (0.94–1.37)	318	338.98	0.94 (0.84–1.05)	235	249.67	0.94 (0.82–1.07)	129	136.56	0.94 (0.79–1.12)
Other diseases of arteries, arterioles, capillaries	71	48.48	1.46^#^ (1.14–1.85)	6	5.85	1.03 (0.38–2.23)	34	20.01	1.70^#^ (1.18–2.37)	20	14.76	1.35 (0.83–2.09)	11	7.85	1.4 (0.7–2.51)
Pneumonia and influenza	290	297.66	0.97 (0.87–1.09)	39	36.46	1.07 (0.76–1.46)	122	123.74	0.99 (0.82–1.18)	76	90.15	0.84 (0.66–1.06)	53	47.31	1.12 (0.84–1.47)
Chronic obstructive pulmonary disease and allied Cond	1360	689.95	1.97^#^ (1.87–2.08)	157	78.38	2.00^#^ (1.7–2.34)	549	279.31	1.97^#^ (1.8–2.14)	426	214.41	1.99^#^ (1.8–2.18)	228	117.85	1.93^#^ (1.69–2.2)
Nephritis, nephrotic syndrome and nephrosis	268	213.48	1.26^#^ (1.11–1.42)	40	24.9	1.61^#^ (1.15–2.19)	93	87.41	1.06 (0.86–1.3)	88	66.26	1.33^#^ (1.07–1.64)	47	34.9	1.35 (0.99–1.79)
Symptoms, signs and ill-defined conditions	176	174.85	1.01 (0.86–1.17)	26	19.13	1.36 (0.89–1.99)	62	69.68	0.89 (0.68–1.14)	60	56.92	1.05 (0.8–1.36)	28	29.12	0.96 (0.64–1.39)
Accidents and adverse effects	275	263.02	1.05 (0.93–1.18)	32	28.7	1.11 (0.76–1.57)	89	103.48	0.86 (0.69–1.06)	97	82.89	1.17 (0.95–1.43)	57	47.94	1.19 (0.9–1.54)
Suicide and self-inflicted injury	12	15.04	0.8 (0.41–1.39)	4	1.88	2.13 (0.58–5.44)	7	6.48	1.08 (0.43–2.23)	0	4.45	0.00^#^ (0–0.83)	1	2.23	0.45 (0.01–2.5)
Other cause of death	1879	2111.31	0.89^#^ (0.85–0.93)	216	224.53	0.96 (0.84–1.1)	644	819.02	0.79^#^ (0.73–0.85)	620	676.59	0.92^#^ (0.85–0.99)	399	391.17	1.02 (0.92–1.13)

*SMR* standardized mortality ratio, *CI* confidence interval

^#^Statistical significance with *P* < 0.05

Data in Table 1 showed subgroup information of female patients undergoing local tumor excision. Most of the deaths were aged 75–84 years, however, compared with the general population, the risk of death was the lowest (SMR 1.53; 95% CI 1.49–1.56). With the decrease of age, the risk level gradually increased, and in the age group of 15–54 years, the risk level was the highest (SMR 3.85; 95% CI 3.58–4.13). White patients compose the majority of deaths, and the risk of death was relatively lower with respect to that of the other three races (*n* = 18,245, SMR 1.77; 95% CI 1.74–1.8). Simultaneously, compared with general population, poorly differentiated and undifferentiated types increased the risk of death by 2.6 and 3.04 times, respectively. The most common pathological types were papillary transitional cell carcinoma and Transitional cell carcinoma (NOS), however, the types that were higher contribution to the risk of death were adenocarcinoma (NOS), small cell carcinoma (NOS), and squamous cell carcinoma (keratinizing, NOS). All year of diagnosis increased the death risk.

### Causes of death for female patients undergoing radical cystectomy

Most deaths for female patients underwent radical cystectomy occurred either 2–11 months (*n* = 1152) or 12–59 months (*n* = 1522) after surgery. Deaths from bladder cancer in this group accounted for 66.9% of all deaths, which composed the majority, while other cancer and non-tumor disease accounted for 10% and 23.1%, respectively (Table 3, Fig. 1). In comparison with the general population, the risk of death in female patients undergoing radical cystectomy significantly increased by 4.67 times over all follow-up months (SMR 4.67; 95% CI 4.51–4.84), which was approximately 2.5 times higher compared with local tumor excision group. Over all follow-up months, the risk of death from bladder cancer was the highest (SMR 832.50; 95% CI 797.86–868.26), and was about 4.4 times higher than that of the local tumor excision group. Simultaneously, several other causes of death were elevated in comparison with the general population, including both other malignant cancers and non-tumor causes. Of all the non-tumor deaths, the most common cause was diseases of heart (n = 195), which account for 25.9% of all non-tumor deaths. The non-tumor cause of death with the highest increased risk of death was septicemia (SMR 3.09; 95% CI 2.13–4.34), and the lowest was Alzheimer’s (SMR 0.64; 95% CI 0.42–0.94).

**Table 3:** Main causes of death for patients with bladder cancer after radical cystectomy

	Total			2–11 months			12–59 months			60–119 months			120 + months		
	Observed	Expected	SMR (95%CI)	Observed	Expected	SMR (95%CI)	Observed	Expected	SMR (95%CI)	Observed	Expected	SMR (95%CI)	Observed	Expected	SMR (95%CI)
All causes of death	3250	695.3	4.67# (4.51/4.84)	1152	88.18	13.06# (12.32/13.84)	1522	268.25	5.67# (5.39/5.97)	375	216.59	1.73# (1.56/1.92)	201	122.27	1.64# (1.42/1.89)
All malignant cancers	2498	142.64	17.51# (16.83/18.21)	973	21.56	45.12# (42.33/48.05)	1291	59.89	21.56# (20.4/22.77)	172	40.77	4.22# (3.61/4.9)	62	20.41	3.04# (2.33/3.89)
Digestive system	38	33.9	1.12 (0.79/1.54)	1	4.97	0.2 (0.01/1.12)	25	14.04	1.78# (1.15/2.63)	8	9.85	0.81 (0.35/1.6)	4	5.05	0.79 (0.22/2.03)
Colon and rectum	16	13.31	1.2 (0.69/1.95)	1	1.97	0.51 (0.01/2.83)	7	5.54	1.26 (0.51/2.6)	5	3.86	1.3 (0.42/3.02)	3	1.94	1.55 (0.32/4.52)
Respiratory system	71	38.46	1.85# (1.44/2.33)	4	6.08	0.66 (0.18/1.68)	30	16.55	1.81# (1.22/2.59)	15	10.73	1.4 (0.78/2.31)	22	5.09	4.32# (2.71/6.54)
Lung and bronchus	70	37.9	1.85# (1.44/2.33)	4	5.99	0.67 (0.18/1.71)	29	16.31	1.78# (1.19/2.55)	15	10.58	1.42 (0.79/2.34)	22	5.02	4.39# (2.75/6.64)
Urinary system	2208	5.4	408.57# (391.7/425.97)	902	0.75	1200.55# (1123.47/1281.52)	1157	2.18	530.82# (500.67/562.31)	125	1.61	77.53# (64.54/92.38)	24	0.86	27.87# (17.86/41.47)
Urinary bladder	2173	2.61	832.50# (797.86/868.26)	887	0.34	2594.47# (2426.51/2770.98)	1143	1.02	1,116.02# (1052.25/1182.64)	119	0.8	148.70# (123.18/177.94)	24	0.44	54.07# (34.64/80.45)
Kidney and renal pelvis	15	2.59	5.79# (3.24/9.54)	7	0.38	18.30# (7.36/37.71)	4	1.08	3.72# (1.01/9.52)	4	0.75	5.33# (1.45/13.64)	0	0.38	0 (0/9.61)
Miscellaneous malignant cancer	138	10.67	12.93# (10.86/15.28)	59	1.56	37.73# (28.72/48.67)	62	4.41	14.07# (10.79/18.04)	12	3.1	3.87# (2/6.76)	5	1.6	3.12# (1.01/7.29)
Non-tumor deaths															
Septicemia	33	10.69	3.09# (2.13/4.34)	18	1.43	12.60# (7.47/19.92)	7	4.23	1.65 (0.66/3.41)	4	3.24	1.23 (0.34/3.16)	4	1.78	2.24 (0.61/5.74)
Diabetes mellitus	20	19.37	1.03 (0.63/1.59)	9	2.83	3.18# (1.46/6.04)	3	7.97	0.38 (0.08/1.1)	5	5.64	0.89 (0.29/2.07)	3	2.94	1.02 (0.21/2.99)
Alzheimer’s	26	40.33	0.64# (0.42/0.94)	3	3.68	0.81 (0.17/2.38)	7	13.37	0.52 (0.21/1.08)	8	13.86	0.58 (0.25/1.14)	8	9.41	0.85 (0.37/1.68)
Diseases of heart	195	173.52	1.12 (0.97/1.29)	39	21.6	1.81# (1.28/2.47)	70	66.59	1.05 (0.82/1.33)	49	54.56	0.9 (0.66/1.19)	37	30.77	1.2 (0.85/1.66)
Cerebrovascular diseases	44	48.14	0.91 (0.66/1.23)	11	5.97	1.84 (0.92/3.3)	10	18.38	0.54 (0.26/1)	18	15.06	1.2 (0.71/1.89)	5	8.72	0.57 (0.19/1.34)
Pneumonia and influenza	25	16.82	1.49 (0.96/2.19)	7	2.04	3.43# (1.38/7.06)	8	6.47	1.24 (0.53/2.44)	6	5.35	1.12 (0.41/2.44)	4	2.96	1.35 (0.37/3.46)
Chronic obstructive pulmonary disease and allied Cond	77	45.98	1.67# (1.32/2.09)	5	6.15	0.81 (0.26/1.9)	28	18.26	1.53# (1.02/2.22)	28	13.98	2.00# (1.33/2.9)	16	7.6	2.11# (1.2/3.42)
Nephritis, nephrotic syndrome and nephrosis	27	13.57	1.99# (1.31/2.89)	4	1.73	2.31 (0.63/5.92)	9	5.28	1.71 (0.78/3.24)	8	4.22	1.9 (0.82/3.74)	6	2.34	2.56 (0.94/5.58)
Symptoms, signs and ill-defined conditions	18	9.52	1.89# (1.12/2.99)	4	1	4.02# (1.1/10.29)	4	3.43	1.17 (0.32/2.98)	6	3.3	1.82 (0.67/3.96)	4	1.8	2.23 (0.61/5.71)
Accidents and adverse effects	23	16.29	1.41 (0.9/2.12)	5	1.99	2.51 (0.82/5.87)	8	6.13	1.3 (0.56/2.57)	3	5.15	0.58 (0.12/1.7)	7	3.02	2.32 (0.93/4.78)
Other cause of death	188	123.69	1.52# (1.31/1.75)	49	13.57	3.61# (2.67/4.77)	55	44.51	1.24 (0.93/1.61)	46	40.86	1.13 (0.82/1.5)	38	24.76	1.53# (1.09/2.11)

*SMR* standardized mortality ratio, *CI* confidence interval

^#^Statistical significance with *P* < 0.05

Data for subgroups of female patients undergoing radical cystectomy can be found in Table 1. The most common death was the 65–74 age group (*n* = 1089), while compared with the general population, the highest risk of death was the 15–54 age group (SMR 19.11; 95% CI 17.22–21.15). Simultaneously, the risk of death obviously increased in all races, and notably, the risk was most elevated in the American Indian/Alaska native group (SMR 19.42; 95% CI 9.69–34.74). The risk of death was similar among different differentiation groups in comparison with the general population, and in different pathological type groups, the transitional cell carcinoma (NOS) composed the majority (*n* = 1813), however, the highest risk was transitional cell carcinoma (spindle cell) (SMR 10.05; 95% CI 7.8–12.74).

## Discussion

In United States, more than 80,500 cases were diagnosed as bladder cancer in 2019 year, which accounted for 4.6% of all cancer diagnoses [12]. Simultaneously, although women are at lower risk of bladder cancer than men, they should be taken seriously. At present, most studies focus on the overall prognosis after diagnosis of bladder cancer, however, reports on the prognosis and cause of death of patients who have undergone different surgical treatment were limited. There are significant differences between radical cystectomy and local tumor excision, including operative area and operative procedures [13]. For radical cystectomy, three options are available, including open radical cystectomy, traditional and robotic laparoscopy. Open radical cystectomy is considered to be the gold standard because of the stably long-term oncological outcomes, however, the characteristics of long time consuming, more blood loss, greater trauma, slow postoperative recovery and high complication rate make people strive for a more minimally invasive surgical method. Traditional laparoscopy can effectively decrease these perioperative risks because of minimally invasive approaches, nevertheless, four degrees of freedom of movement and poor ergonomics caused problems for surgeons. Compared with traditional laparoscopy, robotic surgery is characterized by the wider and clearer vision and more accurate and flexible control capability, but the high surgical cost and long learning curve make it controversial. The long-term oncological outcomes of the minimally invasive surgical methods are still under study [14–17]. A previous study [16] that involved 60 patients suggested that minimally invasive approaches could reach similar oncological outcomes to the open radical cystectomy by comparing the five-year recurrence-free survival, cancer-specific survival and overall survival of patients with bladder cancer who underwent different surgical methods. Moreover, the pathological types of bladder cancer are complex. These factors directly affect the economic burden, spiritual stress, quality of life and prognosis of patients. Hence, this emphasizes the requirements to optimize the selection of surgical methods and health management during survivorship. In our study, we assessed the cause of death after two surgical treatments of bladder cancer stratified by patient and tumor characteristics using representative population-based data from the United States. In female patients undergoing local tumor excision, approximately 50% death from non-tumor causes and 13.8% death from other malignant cancers, however, these women were overall less likely to die of most non-bladder cancer causes in comparison with the general population. In women undergoing radical cystectomy, nearly 82.2% of deaths occurred in 5 years after surgery, and compared with general population, the death of risk caused by non-bladder cancer significantly increased.

Patients with cancer usually have various comorbidities, and the status can directly affect the treatment decision-making, prognosis, and survival outcomes. It is reported that the severity of comorbidity status has a strong impact on the survival of patients in a dose-dependent fashion independent of cancer stage. Coexisting diseases can significantly increase the risk of the mortality of bladder cancer, and the influence degree of individual comorbidities and combined comorbidity is different. Simultaneously, the frequency and severity of perioperative complications increase with comorbidity rates increasing [18–20]. In our study, although the risk of death from heart diseases in all female patients who underwent surgery was slightly higher than that in the general population, it was the most common cause of death. Simultaneously, the ratio of cardiac death was continuously higher than the general population over all follow-up years after the surgery. According to the National Vital Statistics System statistics, 23.4% of the total United States population died of heart diseases in 2015 [21]. Considering these results, death caused by cardiovascular events should be concerned and relative risk factors should be monitored early, such as hyperlipidemia, cigarette smoking, and diabetes mellitus [22]. In patients who underwent radical cystectomy, the risk of death from septicemia was significantly increased in comparison with general population over all follow-up years. Nearly 2/3 of patients occur complications within 90 days after radical cystectomy, and the mortality rate ranges between 1.5% and 2% at 30 days postoperatively [20, 23]. Approximately 25% of the complications are infection, and obstruction caused by ureteral mesenteric anastomosis stenosis and urinary retention can lead to hydronephrosis, renal insufficiency and recurrent urinary tract infection [20]. Therefore, in the management of patients undergoing cystectomy, many long-term sequelae of urinary diversion should be considered, and the nursing of fistula, electrolyte balance and vitamin B12 should be monitored regularly [13]. The choice of the type of urinary diversion is crucial to the quality of life and prognosis of patients undergoing radical cystectomy. Failure of the urinary diversion may lead to the above-mentioned multiple complications and ultimately threaten the life of patients. The ideal urinary diversion should optimally maintain renal function, control urinary outflow, and minimize the incidence rate of patients. Among three types of urinary diversion, including orthotopic neobladders, cutaneous diversions and Ileal conduits, ileal conduits are considered to be the fastest, easiest, least complication-prone urinary diversion [24].

For patients with bladder cancer, age is considered to be an important prognostic factor. Compared with young patients, the mortality rate of elderly patients is higher because of poor histologies, higher recurrence rate, long-term accumulation of the molecular and genetic aberrations, accompanied by comorbidities and decreased immunity [25]. However, for patients undergoing radical cystectomy, it is reported that age is an important prognostic factor but is not irreplaceable, and tumor stage, grade and comorbidity status play decisive roles [26]. Our study showed that the risk of postoperative death in the 15–54 and 55–64 age groups, especially in the 15–54 age group, was significantly higher than that both in the other age groups and in the general population. This result seems different from previous studies, which believe that in contrast to those that occur in older patients, individuals under the age of 40 tend to express well-differentiated histologies and behave in a more indolent fashion [27, 28]. However, previous studies have not updated, and conducted detailed studies on patients after bladder cancer surgery. Young women who underwent surgical treatment, especially cystectomy, have high aggressive and rare pathological types and poor prognosis. However, further research is needed. Simultaneously, the risk of postoperative death in all races was higher than that in the general population, however, the risk of death in non-white patients was obviously higher. Previous study [25] suggested that compared to white females, fewer disease of African Americans with bladder cancer confine to the bladder, and present highly invasive, which may result from socioeconomic status, occupational exposures, smoking, and differences in metabolism of toxic substances. Moreover, in addition to the primary bladder cancer, the death caused by other malignant cancers should also raise concern. The change of hormone level in female patients after operation, or the subsequent treatment, including chemotherapy and immunotherapy, will make the patients in a low immune status, and the combination of other malignant cancers will significantly increase the mortality of patients, especially those who have undergone radical cystectomy [29–31].

The prognosis of patients with bladder cancer is relatively poor, especially women. Female patients are usually diagnosed with more advanced tumors at presentation and have less satisfactory outcomes after treatment with higher cancer-specific mortality. Therefore, multimodal management strategies play important roles in the survival and prognosis of bladder cancer patients, which require the cooperation of multidisciplinary teamwork to take charge of the whole process management of bladder cancer patients, including urology, radiotherapy, oncology, pathology, imaging, nuclear medicine, intervention, anesthesia, nursing and psychotherapy. In addition, personalized treatment and follow-up strategies for different individuals also play an irreplaceable role in multimodal management, including the selection of surgical approaches, the choice of radiotherapy, chemotherapy and immunotherapy at different stages, the improvement of perioperative surgical management, molecular-based systemic treatment strategies, accurate tumor burden assessment, and optimized follow-up policies. Moreover, the progress of molecular tumor biology, the modern research of tumor metastasis, and the development of different approaches has the potential to improve substantially the oncological outcomes [32, 33].

Despite the useful findings of our study, several limitations in our study are as follows: first, some important data lost due to lack of collection in SEER, such as smoking, which has been proved to be a risk factor for bladder cancer prognosis [34]. In addition, this study was based on the classification of surgical methods, which enables us to understand the role of surgical modality in the long-term survival of bladder cancer. However, the non-surgical treatment of bladder cancer is also important for the prognosis of patients. Moreover, different surgeons may have respective treatment strategies for bladder tumors of the same grade. The option of treatment and follow-up methods based on the surgeon's judgment of the final results and the choice of the type of technique proposed will directly affect the prognosis of patients. Finally, the retrospective nature of the SEER database used in the study may have, to an extent, weaken the conclusion.

In summary, this study provides contemporary and comprehensive evaluation of causes of death for female patients of bladder cancer who have underwent radical cystectomy or local tumor excision. We found that the overall risk of death significantly increased for female patients undergoing radical cystectomy or local tumor excision in comparison to the general population, and especially in patients undergoing radical cystectomy. Simultaneously, bladder cancer remains the leading cause of death after surgery, but the death caused by heart diseases could not be ignored, and for patients undergoing radical cystectomy, the death of risk caused by non-bladder cancer significantly increased compared with patients undergoing local tumor excision, such as septicemia. These data highlight the need for general primary care for these female patients during postoperative cancer survivorship.

## Data Availability

The data sets generated during and analyzed during the current study are available in the SEER repository (https://seer.cancer.gov/).
